# Current status and perspectives in translational biomarker research for PD-1/PD-L1 immune checkpoint blockade therapy

**DOI:** 10.1186/s13045-016-0277-y

**Published:** 2016-05-27

**Authors:** Weijie Ma, Barbara M. Gilligan, Jianda Yuan, Tianhong Li

**Affiliations:** Division of Hematology & Oncology, Department of Internal Medicine, University of California Davis Comprehensive Cancer Center, University of California, Davis, School of Medicine, 4501 X Street, Suite 3016, Sacramento, CA 95817 USA; VA Northern California Health Care System, 10535 Hospital Way, Mather, CA 95655 USA; Former visiting medical student from School of Medicine, Peking University Health Science Center, No. 38 Xueyuan Road, Beijing, 100191 China; Immune Monitoring Core, Ludwig Center for Cancer Immunotherapy, Memorial Sloan Kettering Cancer Center, 1275 York Ave, Box 386, New York, NY10065 USA; Present address: Oncology Clinical Research, Merck Research Laboratories, Rahway, NJ07065 USA

**Keywords:** Cancer immunotherapy, Cytotoxic T cells, Immune checkpoint blockade antibodies, PD-1, PD-L1, Immune-related adverse events, Biomarker, Precision oncology

## Abstract

Modulating immune inhibitory pathways has been a major recent breakthrough in cancer treatment. Checkpoint blockade antibodies targeting cytotoxic T-lymphocyte antigen 4 (CTLA-4) and programed cell-death protein 1 (PD-1) have demonstrated acceptable toxicity, promising clinical responses, durable disease control, and improved survival in some patients with advanced melanoma, non-small cell lung cancer (NSCLC), and other tumor types. About 20 % of advanced NSCLC patients and 30 % of advanced melanoma patients experience tumor responses from checkpoint blockade monotherapy, with better clinical responses seen with the combination of anti-PD-1 and anti-CTLA-4 antibodies. Given the power of these new therapies, it is important to understand the complex and dynamic nature of host immune responses and the regulation of additional molecules in the tumor microenvironment and normal organs in response to the checkpoint blockade therapies. In this era of precision oncology, there remains a largely unmet need to identify the patients who are most likely to benefit from immunotherapy, to optimize the monitoring assays for tumor-specific immune responses, to develop strategies to improve clinical efficacy, and to identify biomarkers so that immune-related adverse events can be avoided. At this time, PD-L1 immunohistochemistry (IHC) staining using 22C3 antibody is the only FDA-approved companion diagnostic for patients with NSCLC-treated pembrolizumab, but more are expected to come to market. We here summarize the current knowledge, clinical efficacy, potential immune biomarkers, and associated assays for immune checkpoint blockade therapies in advanced solid tumors.

## Background

Since 2011, when the US Food and Drug Administration (FDA) approved ipilimumab for advanced melanoma, there has been an explosion of research interest and drug development for harnessing the immune system to fight against cancer. A general search for “immune therapy and cancer” in the clinicaltrials.gov database from 2000 to 2010 shows 640 hits, while the same search on March 20, 2015 shows 7815 hits and December 23, 2015 shows 8941 hits. Active research in tumor immunology includes studies on adoptive T cell therapy and cancer vaccine, as well as clinical investigations into immune checkpoint blockade in monotherapy or combination therapies. First generation programed cell-death protein 1 (PD-1) blockade antibodies pembrolizumab and nivolumab obtained US FDA approval for advanced melanoma in 2014 and as second line therapy for non-small cell lung cancer (NSCLC) in 2015. We have seen a surge in the availability of immune checkpoint inhibitors for multiple cancer indications. Forecasts for 2013–2020 project that the market for immunotherapy will increase from approximately $1 billion (US dollars) in 2013 to in excess of $7 billion in 2020 (corresponding to 33 % annual growth), across the seven major markets (USA, France, Germany, Italy, Spain, UK, and Japan) [1].

Unlike chemotherapy and molecularly targeted therapy, the checkpoint blockade immunotherapies result in durable clinical responses through the induction, activation, and expansion of tumor-specific cytotoxic T cells. Immune checkpoints play an essential role in maintaining self-tolerance and regulating the amplitude and duration of T cell responses. Two cytotoxic T cell immune checkpoint receptors (cytotoxic T-lymphocyte antigen 4 (CTLA-4) and PD-1) deliver inhibitory signals when bound to their respective ligands CD80/86 and programed cell-death ligand 1 (PD-L1)/2. Immunotherapies with checkpoint blockade antibodies that block CTLA-4 and PD-1 (or its ligand PD-L1) can restore and augment cytotoxic T cell responses against chemotherapy-refractory tumors, leading to durable responses and prolonged overall survival with tolerable toxicity. In the lymphatic tissues, anti-CTLA-4 antibody induces non-tumor-specific immune activation that is largely de novo induction of new responses. In tumor microenvironment, anti-PD-1/PD-L1 antibody targets tumor-induced immune defects and repairing ongoing tumor immunity. In addition to these anti-CTLA-4 and anti-PD-1/PD-L1 antibodies, drugs targeting other immune checkpoints and/or co-stimulatory ligand-receptor inhibitors, such as lymphocyte activation gene 3 protein (LAG3) and T cell immunoglobulin and mucin-domain containing protein 3 (TIM-3), are currently being explored in many tumor types and will be discussed further in the “Principles of cancer immunotherapy” section.

Given the distinct and novel mechanisms of action of immunotherapy on effector immune cells, rather than tumor cells, there are emerging tumor response patterns that are not accurately assessed by Response Evaluation Criteria in Solid Tumors (RECIST). In 2009, a guideline for immune-related response criteria (irRC) was proposed for patients with advanced melanoma receiving ipilimumab [2]. This guideline has been used to evaluate the tumor response for ongoing immune checkpoint blockade therapies [3]. When assessed appropriately, approximately 20 % of advanced NSCLC patients and 30–40 % of advanced melanoma patients have objective tumor responses to PD-1 blockade monotherapy. The combination of anti-PD-1 and anti-CTLA-4 antibodies increased the clinical response in advanced melanoma patients with a recent FDA approval [4]. In an attempt to increase the therapeutic, in checkpoint inhibitors, a companion diagnostic of PD-L1 immunohistochemistry (IHC) had been approved by the US FDA for selecting NSCLC patients treated with pembrolizumab. Interestingly, overall tumor responses, progression-free survival (PFS) and overall survival (OS) were not consistently associated with PD-L1 expression in the patients receiving immune checkpoint inhibitors [5]. In this age of personalized medicine, finding and validating better immune biomarkers will enable us to select patients for cancer immunotherapies. It will also lead to a better understanding of the unique mechanisms of tumor response and resistance, to allow for the development of strategies to improve clinical efficacy, and to lead to better evaluation of response and monitoring assays for host immune function. Validating potential immune biomarkers will enable us to better select patients for cancer immunotherapies and to avoid immune-related adverse effects (irAEs). Many excellent review articles on cancer biomarkers and immune checkpoint inhibitors have been recently published [6–12]. In this review, we will focus on the current knowledge, clinical efficacy, available monitoring assays, and emerging novel technologies for identifying translational biomarkers for immune checkpoint blockade immunotherapies in advanced solid tumors.

## Principles of cancer immunotherapy

Clinically apparent cancer can be thought of as a host’s inability to eliminate transformed cells. Cancer immunotherapy refers to a diverse range of therapeutic approaches that harness the immune system to induce or restore the capacity of cytotoxic T cells, and other immune effector cells, and to recognize and eliminate cancer [13, 14]. The field of cancer immunotherapy is undergoing a renaissance due to a greater understanding of the immune system and immunosurveillance. The immune system protects the host against tumor development but also promotes tumor growth by selecting for tumors of lower immunogenicity. This dual effect of the immune system on developing tumors creates a dynamic process termed *cancer immunoediting*, which is comprised of three phases: elimination, equilibrium, and escape [15]. Cancer cells are immunogenic through the generation of tumor-specific mutant antigens (TSMA, neoantigens) from somatic gene structural and/or epigenetic alterations [16]. During the *elimination phase*, immune effector cells (mainly T and natural killer (NK) lymphocytes) are activated by the inflammatory cytokines released by the growing tumor cells, macrophages, and stromal cells surrounding the tumor cells. The recruited tumor-infiltrating NK cells and macrophages produce interleukin 2 (IL-2), interleukin 12 (IL-12), and interferon gamma (IFNγ), which kill tumor cells by cytotoxic mechanisms such as perforin, tumor necrosis factors (TNF)-related apoptosis-inducing ligands (TRAILs), and reactive oxygen species. Due to heterogeneity, tumor cells with a less immunogenic phenotype are able to escape this elimination phase (i.e., immunosurveillance) and expand during the *equilibrium phase*. The equilibrium phase may occur over a period of many years when, under Darwinian selection, new tumor cell variants emerge with mutations that further increase overall resistance to immune attack. During the *escape phase*, tumor cell variants breach the host’s immune defenses conferring further resistance to immune detection [17].

Major mechanisms for *escape* include defective antigen presentation, tumor-induced inhibitory checkpoint pathways against effector T cell activity, infiltrating immunosuppressive immune cells including regulatory T cells (Treg) and myeloid-derived suppressor cells (MDSCs), and secretion of immunosuppressive cytokines (transforming growth factor beta (TGF-β), IL-6, vascular endothelial growth factor (VEGF)) [7, 18, 19]. Full activation of T and NK lymphocytes requires the coordinated participation of several surface receptors that meet their cognate ligands through structured transient cell-to-cell interactions known as immune synapses. Current PD-1/PD-L1 blockade therapies aim to boost the patient’s effector T cells to specifically recognize and kill cancer cells. Table 1 summarizes the current list of major immune checkpoint inhibitors that have obtained FDA approval or are in the late phases of clinical development.

**Table 1:** Summary of clinical indication and ongoing evaluation of immune checkpoint inhibitors in major cancer types

Target	Drug	Class	Company	Clinical indication and ongoing evaluation (status; approval date; trial identifier; country)
CTLA-4	Ipilimumab (Yervoy®, MDX-010, MDX-101)	Human IgG1/kappa	Bristol-Myers Squibb	*Metastatic melanoma* (US FDA approved on March 25, 2011); *metastatic NSCLC* (phase I has been completed; NCT01165216; Japan; phase II reported, NCT00527735, USA; phase III ongoing, NCT01285609; USA; phase III ongoing NCT02279732; China)
	Tremelimumab (ticilimumab, CP-675206)	Human anti-CTLA-4 IgG2 mab	MedImmune/AstraZeneca	*Metastatic melanoma* (phase I has been completed; NCT01103635; USA; phase II has been completed, NCT00471887, USA); *advanced hepatocellular carcinoma* (phase II has been completed; NCT01008358; Spain); *Metastatic NSCLC* (phase Ib has been reported, NCT02000947, USA; phase II has been reported, NCT02179671, USA; first line, phase III MYSTIC ongoing; NCT02453282; USA)
PD-1	Nivolumab (Opdivo®, ONO-4538, MDX-1106, BMS-936558)	Human IgG4/kappa	Bristol-Myers Squibb; Ono Pharmaceuticals	*Metastatic melanoma* (Japan approval on July 4, 2014; US FDA accelerated approval on December 22, 2014; US FDA approval of nivolumab in combination with ipilimumab for BRAF V600 wild-type tumor on September 30, 2015); *Squamous NSCLC* (US FDA approval on March 4, 2015; European Commission on July 20, 2015); expands to *non-squamous NSCLC* (US FDA approval on October 9, 2015); *advanced (metastatic) renal cell carcinoma* (US FDA approval on November 23, 2015); classical Hodgkin lymphoma that has relapsed or progressed after autologous hematopoietic stem cell transplantation and post-transplantation brentuximab vedotin (US FDA approval on May 17, 2016)
	Pembrolizumab (Keytruda®, lambrolizumab, MK-3475)	Humanized IgG4	Merck & Co.	*Metastatic melanoma* (USA accelerated approval on September 4, 2014 for patients with disease progression after ipilimumab and, if BRAF V600 mutation positive, a BRAF inhibitor; US FDA expanded to initial treatment on December 18, 2015); *metastatic NSCLC* whose tumors express PD-L1 as determined by an FDA-approved test and who have disease progression on or after platinum-containing chemotherapy (US FDA approval on October 2, 2015)
	Pidilizumab (CT-011)	Humanized IgG1	CureTech Ltd	*Diffuse large-B cell lymphoma* (phase II has been completed; NCT00532259; USA); *metastatic melanoma* (phase II has been completed; NCT01435369; USA)
	AMP-514 (MEDI0680)	Humanized IgG4	MedImmune	*Advanced malignancies* (phase II is currently recruiting participants; NCT02013804; USA)
	AUNP-12	Peptide antagonist	Aurigene, Pierre Fabre	*Cancer* (preclinical phase, Aurigene granted Pierre Fabre worldwide rights to develop AUNP12 for cancer indications; announced on February 11, 2014; India)
PD-L1	BMS936559 (MDX-1105)	Human IgG4	Bristol-Myers Squibb	*Advanced or recurrent solid tumors* (phase II has been completed; NCT00729664; USA)
	Atezolizumab (Tecentriq™, MPDL3280A, RG7446)	Human IgG1	Roche & Genentech	*Metastatic bladder cancer* (phase III, US FDA granted breakthrough therapy designation on May 31, 2014; priority review on March 14, 2016; accelerated approval on May 18, 2016); *metastatic NSCLC* (phase III, US FDA grants breakthrough therapy designation on February 1, 2015)
	Durvalumab (MEDI4736)	Humanized IgG1	AstraZeneca	*Glioblastoma* (phase II is currently recruiting participants; NCT02336165; USA); *metastatic squamous cell carcinoma of the head and neck* (phase II is currently recruiting participants; NCT02207530; USA); *advanced or metastatic NSCLC* (phase III is currently recruiting participants; NCT02352948; Global study); *advanced colorectal cancer* (phase II is currently recruiting participants; NCT02227667; USA); *metastatic NSCLC* (first line phase III MYSTIC is current recruiting participants; NCT02453282; USA; first line phase III ARCTIC is current recruiting participants; NCT02352948; Global); *metastatic bladder cancer* (US FDA granted breakthrough therapy designation for PD-L1-positive tumors in patients who progressed during or after one standard platinum-based regimen on February 17, 2016)
	Avelumab (MSB0010718C)	Fully humanized IgG1	Merck KGaA, EMD Serono, Pfizer	*Advanced solid tumors* (phase I with consecutive parallel group expansion; currently recruiting participants in multiple tumor types and settings; NCT01772004; USA); *metastatic NSCLC* (phase III is currently recruiting participants after failure of a platinum-based doublet; NCT02395172; and first line versus platinum doublet; NCT02576574; USA)
PD-L2	AMP-224	PD-L2-IgG2a fusion protein	Amplimmune	*Advanced cancer* (phase II has been completed; NCT01352884; USA)

Last assessed information at ClinicalTrial.gov on December 28, 2015; updated FDA approvals on May 18, 2016. Italicized data highlights major cancer types in clinical evaluation

*Abbreviations*: *CTLA-4* cytotoxic T-lymphocyte-associated protein 4, *NSCLC* non-small-cell lung cancer, *PD1* programed cell-death protein 1, *PDL1* programed cell-death ligand 1

Immune checkpoints normally maintain self-tolerance, regulate the amplitude and duration of T cell responses, and prevent tissue damage. PD-1 and PD-L1 immune checkpoint blockade can restore the function of exhausted CD8 T cells in chronic viral infection, augmenting the existing tumor-specific immunity [20, 21]. Nevertheless, immune checkpoint inhibitors can override immune self-tolerance, inducing a unique syndrome of autoimmune and autoinflammatory side effects (i.e., irAEs) [22]. Many of these irAEs resemble autoimmune diseases, such as autoinflammatory rheumatic disease, dermatologic disease, colitis, hepatitis, and endocrinopathies [23]. Therefore, almost all immunotherapy clinical trials have excluded individuals with preexisting autoimmune diseases, although a recent study demonstrates that the use of ipilimumab in cancer patients with autoimmune diseases is safe and effective [24]. However, a recent study showed the safety and efficacy of using anti-CTLA-4 antibody in patients with autoimmune disease [24]. Early detection and effective management of these irAEs are pivotal for a favorable clinical outcome. Theoretically, cytotoxic T cells against tumors and normal organs are different subclones that may not need to be activated in parallel. Further studies are warranted to specifically enhance tumor-specific cytotoxic T cell response and avoid unnecessary T cell response to normal tissues.

## Biology and mechanisms of PD-1 and PD-L1 molecules

PD-1 (CD279) is expressed on many cell types including T cells, B cells, natural killer T cells, activated monocytes, and dendritic cells (DCs) in humans and mice [25]. In normal human lymphoid tissues, PD-1 is detected on germinal center-associated T cells [26]. PD-1 binds to the ligand PD-L1 (B7-H1, CD274) or PD-L2 (B7-DC, CD272). PD-L1 is typically expressed on the surface of tumor cells [27]. PD-L1 is also constitutively expressed on other cell types such as T and B cells, DCs, macrophages, mesenchymal stem cells, and bone marrow-derived mast cells [28]. Cell surface expression of PD-L1 can be upregulated on both tumor cells and other cell types after treatment with type I or type II interferons (IFNs) [29, 30], radiation [31–33], or chemotherapy [34–38]. Additionally, radiotherapy may induce direct tumor-cell killing and multiple immunomodulatory changes that can potentially influence the effectiveness of immunotherapy [31–33].

Engagement of PD-1 by its ligands, either PD-L1 or PD-L2, induces a negative control signal resulting in the inhibition of T cell proliferation, cytokine production, and cytotoxic activity. PD-L1 can provide inhibitory signals to activated T cells through interactions with the surface receptor PD-1 or CD80 [25, 39]. These signals enable PD-L1-expressing tumor cells to evade immune detection by NK cells or T cells [40–42]. PD-L2 is encoded by PDCD1LG2, which is essential for T cell proliferation and IFN production. PD-L2 can stimulate PD-1 receptor signaling, resulting in “T cell exhaustion,” a temporary inhibition of activation and proliferation that can be reversed by the removal of PD-1 signal [25].

**Fig. 1:**
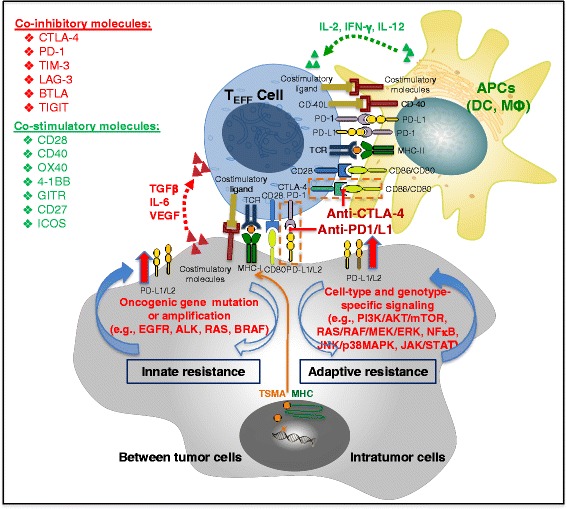
Schema interaction between tumor and immune cells. Full activation of T-lymphocytes requires the coordinated participation of several surface receptors on effector T cells and antigen-presenting cells (APCs) or tumor cells. The main route of T cell stimulation is driven by antigens recognized in the form of short polypeptides associated with MHC antigen-presenting molecules. However, the functional outcome of T cell stimulation towards clonal expansion and effector function acquisition is contingent on the contact of additional surface receptor-ligand pairs and on the actions of cytokines in the tumor microenvironment. While some of those interactions are inhibitory (in *red*), others are activating and are collectively termed co-stimulatory (in *green*) receptors. Communication between T cells and APCs is bidirectional. In some cases, this occurs when ligands themselves signal to the APC. In other cases, activated T cells upregulate ligands, such as CD40L, that engage cognate receptors on APCs. Tumor cells can upregulate PD-L1 expression via either the constitutionally activated oncogenic signaling (*left*, innate/intrinsic immune resistance) or the immune modulator-induced signaling pathways (*right*, adaptive immune resistance). *Abbreviations*: *APC* antigen-presenting cells, *DC* dendritic cell, *IL-2R* IL-2 receptor, *MDSCs* myeloid-derived suppressor cells, *Teff* effector T cell, *Treg* regulatory T cells, *IDO* indoleamin 2,3-dioxygenase, *TIM-3* T cell immunoglobulin domain and mucin domain, *LAG* lymphocyte-activation gene, *BTLA* B- and T-lymphocyte attenuator, *HVEM* herpes virus entry mediator, *TIGIT* T cell immunoreceptor with Ig and ITIM domains, *GITR* glucocorticoid-induced tumor necrosis factor receptor, *ICOS* inducible costimulators, *CEACAM* carcinoembryonic antigen-related cell adhesion molecule, *TSMA* tumor-specific mutant antigens, *JNK*, c-Jun N-terminal kinase, *MEK/ERK*, mitogen/extracellular signal regulated kinase, *PI3K*, phosphatidylinositol 3-kinase, *STAT*, signal transducer and activator of transcription, *NFκB*, nuclear factor kappa-light-chain-enhancer of activated B cells

Upregulation of PD-L1 on different tumor types inhibits the local antitumor T cell response. Figure 1 illustrates the expression and complex interaction of key molecules and co-stimulatory ligand receptors between tumor and immune cells in tumor microenvironment (TME), and the two potential mechanisms proposed to understand the biology and function of PD-L1 on tumor cells [43]. While constitutive oncogenic signals (such as protein kinase B (AKT), epidermal growth factor receptor (EGFR), KRAS pathways) upregulate PD-L1 expression on tumors as innate (tumor cell intrinsic) resistance (Fig. 1, left) [44–48], inflammatory signals generate cytokine-induced PD-L1 expression on either tumor cells or immune cells (myeloid suppressor cells, dendritic cell, macrophage, and lymphocytes) in the tumor microenvironment as adaptive resistance (Fig. 1, right) [49, 50]. Preclinical studies have demonstrated that blocking the PD-L1/PD-1 interaction increases the numbers of effector T cells, augments cytolytic activity of tumor-specific T cells, enhances the production of pro-inflammatory cytokines, brings effector T cells to the tumor sites, reduces numbers and suppression of Tregs at the tumor site, and downregulates the production of suppressive cytokines IL-10 [51–54]. Tumor-infiltrating T cells may also be functionally inert, due in part to the expression of PD-1 along with other inhibitory receptors [27, 55]. The hallmarks of the PD-1/PD-L1 blockage effect include immune modulation at tumor site, direct targeting of tumor-induced immune defects, and repairing ongoing tumor immunity. PD-1 deletion in mice can lead to autoimmunity, most notably when bred onto backgrounds of autoimmune-susceptible mouse strains [56, 57]. In multiple syngeneic mouse tumor models, blockade of PD-1 or its ligands promotes antitumor activity [58, 59]; anti-PD-1 activity in vivo can be enhanced by combination with antibodies to other T cell-negative regulators, such as CTLA-4 and LAG-3 [60–62]. A recent study suggests that PD-L1 is an independent prognostic marker in melanoma, defining a tumor subset with distinct genetic and morphophenotypic features [63].

## Clinical efficacy of PD-1/PD-L1 blockade therapies and PD-L1 expression

Nivolumab and pembrolizumab are the first generation PD-1 immune checkpoint blockade antibodies. Nivolumab inhibits the interaction between PD-1 and its ligands, PD-L1, and PD-L2 with similar IC_50_ values (2.52 and 2.59 nmol/L, respectively). Nivolumab enhances T cell reactivity in the presence of a T cell receptor stimulus at the low concentration (~1.5 ng/mL) without nonspecific lymphocyte activation. Nivolumab also enhances the production of inflammatory cytokines in assays such as the human T cell/DC-mixed lymphocyte reaction (MLR) assays, and Staphylococcal enterotoxin B (SEB), and cytomegalovirus (CMV) recall response assays. In addition, nivolumab increases antigen-specific CD8^+^ T cell responses in patients with melanoma [64]. Nivolumab completely restored CD4^+^ T-responder cell proliferation and partially restored IFNγ production. Nivolumab also overcomes Treg suppression of CD8^+^ T cells by increasing resistance to Treg suppression and also by directly limiting suppressive capacity of Treg [65]. This was demonstrated in monkeys when increased numbers of CD8^+^ T-effector memory cells were detected in CMV-positive peripheral blood after repeated treatment at the highest dose of nivolumab for 3 months. Similarly, marked accumulation of CD8^+^ effector memory T cells in lymphoid organs and tissues of PD-1-deficient mice has been described [66].

Both nivolumab and pembrolizumab have shown significant survival benefit in some patients with advanced melanoma, NSCLC, and renal cell carcinomas in early clinical trials (Table 1). These agents were generally well tolerated, even in elderly patients with prolonged dosing. Lower tumor response rates have been observed in never smokers and patients with EGFR mutations or ALK rearrangements when compared to heavy smokers. Table 2 summarizes this clinical efficacy data from four of the most studied PD-1 and PD-L1 inhibitors at various stages of clinical development. As only subsets of patients are benefiting from the PD-1/PD-L1 immune checkpoint blockade therapies, it is important to try to select for this population so that patients less likely to improve with therapy can be spared toxicities. An additional challenge is that response criteria used for standard therapy may not be applicable for immunotherapy. Unlike standard therapeutics in which tumor response is measured by a variety of radiographic and laboratory tests every 6–8 weeks, delayed tumor responses due to the time required for activation and function of effector T cells are frequently observed in cancer patients treated with immune checkpoint blockade therapies. In some patients, tumor regression persisted after discontinuation of PD-1 blockade therapy [67]. Evaluating responses appropriately and identifying patients who are most likely to respond are important aspects of immunotherapy that continue to be developed.

**Table 2:** Summary of reported clinical efficacy of PD-1/PD-L1 inhibitors

Agent	Clinical trials identifier	Phase of clinical trial	Sample size (no. Pt)	Patient population	Biomarker	Regimen	Tumor responses (ORR)	Median PFS (months)	OS (months; median unless otherwise specified)	Reference: author (year)
Nivolumab	NCT01642004 (CheckMate 017)	Phase III	272 all: 135 nivolumab, 137 docetaxel	Advanced squamous NSCLC with disease progression during or after first-line chemotherapy	PD-L1-positive tumor cells	Nivolumab 3 mg/kg IV every 2 weeks	20 %	3.5	9.2	Brahmer J (2015) [125]
						Docetaxel 75 mg/m^2^ IV every 3 weeks	9 %	2.8	6	
	NCT01673867 (CheckMate 057)	Phase III	582 all: 292 nivolumab, 290 Docetaxel	Advanced non-squamous NSCLC after platinum-based doublet chemotherapy	PD-L1-positive tumor cells	Nivolumab 3 mg/kg IV every 2 weeks	19 %	2.3	12.2	Borghaei H (2015) [126]
						Docetaxel 75 mg/m2 IV every 3 weeks	12 %	4.2	9.4	
	NCT01668784 (CheckMate 025)	Phase III	821 all: 406 nivolumab, 397 Everolimus	Advanced clear-cell renal-cell carcinoma with one or two regimens of anti-angiogenic therapy	PD-L1-positive tumor cells	Nivolumab 3 mg/kg IV every 2 weeks	25 %	4.6	25	Motzer RJ (2015) [127]
						Everolimus 10 mg orally daily	5 %	4.4	19.6	
	NCT01721746 (CheckMate 037)	Phase III	631 all: 272 nivolumab,133 investigators choice of chemo	Unresectable or metastatic melanoma after ipilimumab or ipilimumab and BRAF inhibitor if BRAF positive	PD-L1-positive tumor cells	Nivolumab 3 mg/kg IV every 2 weeks	32 %	4.7	NA	Weber JS (2015) [128]
						Chemo: either dacarbazine 1000 mg/m2 IV every 3 weeks or carboplatin AUC = 6 plus paclitaxel 185 mg/m2 IV every 3 weeks	11 %	4.2	NA	
	NCT01927419 (CheckMate 069)	Phase III	142 all: 95 nivolumab + ipilimumab, 47 ipilimumab	Unresectable or metastatic melanoma treatment naïve with measurable disease	Tissue available for PD-L1 biomarker analysis	Nivolumab 1 mg/kg IV every 3 weeks × 4 doses plus ipilimumab 3 mg/kg IV every 3 weeks × 4 doses, then maintenance nivolumab 3 mg/kg IV every 2 weeks	BRAF wild type: 61 %; BRAF mutation: 52 %	BRAF wild type: NR; BRAF mutation: 8.5	NA	Postow MA (2015) [4]
						Same dose schedule with nivolumab placebo in both the combination and maintenance phase	BRAF wild type: 11 %; BRAF mutation: 22 %	BRAF wild type: 4.4; BRAF mutation: 2.7	NA	
	NCT01721772 (CheckMate 066)	Phase III	418 all: 210 nivolumab, 208 dacarbazine	Untreated metastatic melanoma without BRAF mutation	Tissue available for PD-L1 biomarker analysis	Nivolumab 3 mg/kg IV every 2 weeks plus placebo every 3 weeks	40 %	5.1	1-year OS: 72.9 %	Robert C (2015) [129]
						Dacarbazine 1000 mg/m2 IV every 3 weeks plus placebo every 2 weeks	13.9 %	2.2	1-year OS: 42.1 %	
	NCT01844505 (CheckMate 067)	Phase III	945 all: 316 nivolumab, 314 combination, 315 ipilimumab	Untreated, unresectable stage III or IV melanoma	Tissue available for PD-L1 biomarker analysis	Nivolumab 3 mg/kg IV every 2 weeks (plus ipilimumab placebo)	43.7 %	6.9	NA	Larkin J (2015) [130]
						Nivolumab 1 mg/kg IV every 3 weeks plus ipilimumab 3 mg/kg IV every 3 weeks × 4 doses; then maintenance nivolumab 3 mg/kg IV every 2 weeks	58 %	11.5		
						Ipilimumab 3 mg/kg IV every 3 weeks (plus nivolumab placebo)	19 %	2.9		
	NCT01721759 (CheckMate 063)	Phase II single arm trial	117	Advanced NSCLC	PD-L1-positive tumor cells	Nivolumab 3 mg/kg every 2 weeks until progression or unacceptable toxic effects	14.5 %	1.9	8.2	Rizvi NA (2015) [131]
	NCT00730639	Phase I with expansion cohorts	107	Advanced melanoma	Unselected	Nivolumab at 1, 3, or 10 mg/kg every 2 weeks for up to 96 weeks	31 %	3.7	16.8	Topalian SL (2014) [67]
		Phase I with expansion cohorts	34	Previously treated advanced RCC	Unselected	Nivolumab at 1, 3, or 10 mg/kg every 2 weeks for up to 96 weeks	29 %	7.3	22.4	McDermott DF (2015) [132]
		Phase II with expansion cohorts	129	Heavily pretreated advanced NSCLC	Unselected	Nivolumab at 1, 3, or 10 mg/kg every 2 weeks for up to 96 weeks	17 %	2.6	9.9	Gettinger SN (2015) [133]
Pembrolizumab	NCT01866319 (KEYNOTE-006)	Phase III	834 all: 279 pembrolizumab, 277 pembrolizumab, 278 ipilimumab	Unresectable stage III or IV melanoma	PD-L1-positive tumor cells	Pembrolizumab 3 mg/kg IV every 2 weeks	33.7 %	5.5	NA	Robert C (2015) [134]
						Pembrolizumab 3 mg/kg IV every 3 weeks	32.9 %	4.1		
						Ipilimumab 3 mg/kg IV every 3 weeks	11.9 %	2.8		
	NCT01704287 (KEYNOTE-002)	Phase II	540 all: 180 pembrolizumab, 181 pembrolizumab, 179 chemotherapy	Ipilimumab-refractory melanoma	Will be reported with the final overall survival analysis	Arm A 2 mg/kg (*n* = 180)	21 %	5.4	NA	Ribas A (2015) [135]
						Arm B 10 mg/kg (*n* = 181)	25 %	5.8		
						Chemotherapy	4 %	3.6		
	NCT012958297 (KEYNOTE-001)	Phase I	495	Advanced NSCLC	PD-L1-positive tumor cells	Pembrolizumab 2 or 10 mg/kg IV every 3 weeks or 10 mg/kg every 2 weeks over a 30-min period	19.4 %	3.7	12	Garon EB (2015) [5]
		Phase I	655	Melanoma	Unselected	Pembrolizumab 2 mg/kg every 3 weeks (Q3W), 10 mg/kg Q3W, or 10 mg/kg Q2W until unacceptable toxicity, disease progression, or investigator decision	33 %	12-month PFS 35 %	23	Adil Daud (2015) [136]; Ribas A (2016) [137]
		Phase I with expansion cohort	173	Advanced melanoma after at least 2 ipilimumab doses	Unselected	Pembrolizumab 2 mg/kg IV every 3 week or 10 mg/kg IV every 3 weeks	26 %	2	NA	Robert C (2014) [138]
	NCT01848834 (KEYNOTE-012)	Phase IB	32	Metastatic triple-negative breast cancer	PDL-1-positive tumor cells	Pembrolizumab 10 mg/kg IV every 2 weeks	19 %	6-month PFS 23.3 %	NA	Nanda R (2014) [139]
	NCT1905657 (KEYNOTE-010)	Phase II/III	1034 all: 339 pembrolizumab, 343 pembrolizumab, 309 docetaxel	Previously treated PD-L1-positive advanced NSCLC	PDL-1-positive tumor cells	Pembrolizumab 2 mg/kg, IV every 3 weeks	18 %	3.9 months	14.9	Herbst RS [2015] [140]
						Pembrolizumab 10 mg/kg, IV every 3 weeks	18.5 %	4.0 month	17.3	
						Docetaxel, 75 mg/m2 every 3 weeks	9.3 %	4.0 month	8.2	
	NCT01953692 (KEYNOTE-013)	Phase IB	15	Hodgkin lymphoma	Unselected	Pembrolizumab 10 mg/kg IV every 2 weeks up to 2 years	53 %	NA	NA	Moskowitz C (2014) [141]
Atezolizumab (MPDL3280A)	NCT01846416	Phase II	205	NSCLC	PD-L1-positive tumor cells	Atezolizumab 1200 mg IV every 3 weeks	The highest ORR was seen in pts with PD-L1 TC3 or IC3 tumors	NA	NA	Spigel DR (2015) [142]
	NCT01903993 (POPLAR)	Phase II	287	Previously treated NSCLC patients (pts) were stratified by PD-L1 IC status	PD-L1-positive tumor cells	Atezolizumab 1200 mg IV every 3 weeks	57 %	2.7	12.6	Spira AI (2015) [143]; Fehrenbacher L (2016)
						Docetaxel 75 mg/m2 IV every 3 weeks	24 %	3.0	9.7	
	NCT01375842	Phase I	35	Metastatic melanoma	PD-L1-positive tumor cells	Atezolizumab IV every 3 weeks for up to 1 year	26 %	24-week PFS 35 %	NA	Omid Hamid (2013) [144]
		Phase I	277	Multiple cancer types	PD-L1-positive tumor cells	Atezolizumab intravenously every 3 weeks doses >1 ml/kg	18 %	2.6	NA	Herbst RS (2014) [145]
	NCT01633970	Phase Ib	37	Untreated NSCLC	PD-L1-positive tumor cells	Atezolizumab 15 mg/kg IV every 3 weeks with standard chemo dosing for 4–6 cycles followed by MPDL3280A maintenance therapy until progression	67 %	NA	NA	Stephen V (2015) [146]
		Phase Ib	14	Arm A: refractory metastatic colorectal cancer; arm B: oxaliplatin-naive mCRC	Not mentioned	Arm A: MPDL3280A 20 mg/kg every 3 weeks and bevacizumab (bev) 15 mg/kg every 3 weeks	8 % (1/13) in arm A	NA	NA	Bendell, J.C. (2015) [147]
						Arm B: MPDL3280A 14 mg/kg every 2 weeks, bev 10 mg/kg every 2 weeks and mFOLFOX6 at standard doses	36 % (9/25) in Arm B	NA	NA	
		Phase Ib	12	Metastatic renal cell carcinoma	Not selected	Atezolizumab 15 mg/kg given alone on cycle 1 day 1 and concurrently with 20 mg/kg every 2 weeks thereafter	40 %	NA	NA	Sznol M (2015) [148]
Durvalumab (MEDI4736)	NCT01693562	Phase I/II	198	NSCLC	Tissue available for PD-L1 biomarker analysis	Durvalumab 10 mg/kg IV every 2 weeks until unacceptable toxicity, disease progression, or for up to 12 months	14 % (23 % in PD-L1+ tumors)	NA	NA	Rizvi NA (2015) [149]
		Phase I	13	NSCLC	Tissue available for PD-L1 biomarker analysis	Durvalumab 7 doses (1–25) across 6 cohorts (0.1–10 mg/kg every 2 weeks; 15 mg/kg every 3 weeks)	NA	NA	NA	Brahmer JR (2014) [150]
		Multi-arm expansion study	62	A squamous cell carcinoma of the head and neck expansion cohort	Tissue available for PD-L1 biomarker analysis	Durvalumab IV every 2 weeks at 10 mg/kg for 12 months	12 % (25 % in PD-L1+ pts)	NA	NA	Segal NH (2015) [151]
		Phase I	26	Advanced solid tumors		Durvalumab IV every 2 (q2w) or 3 weeks (q3w) in a 3 + 3 dose escalation with a 28-day (q2w) or 42-day (q3w) DLT window	NA	NA	NA	Lutzky J (2014) [152]
	NCT02088112	Phase I	10	NSCLC	Unselect	Durvalumab cohort A received 3 mg/kg (starting dose) every 2 weeks plus gefitinib 250 mg QD	NA	NA	NA	Creelan BC (2015) [153]
	NCT02000947	Phase Ib	118 (102 eligible)	NSCLC	Tissue available for PD-L1 biomarker analysis	Durvalumab 10–20 mg/kg every 2 or 4 weeks plus tremelimumab 1 mg/kg (*N* = 56)	23 % (6/26): 22 % (2/9) in PD-L1+ versus 29 % (4/14) in PD-L1-	NA	NA	Antonia SJ (2015) [154]; Updated in Antonia SJ (2016) [155]
						Durvalumab 10–20 mg/kg every 2 weeks plus tremelimumab 3 mg/kg (*N* = 34)	20 % (5/25)			
						Durvalumab 15 mg/kg every 4 weeks plus tremelimumab 10 mg/kg (*N* = 9)	0 % (0/9)			

*Abbreviations*: *DLT* dose-limiting toxicity, *q2w* every 2 weeks, *q3w* every 3 weeks, *q4w* every 4 weeks, *QD* once daily, *BRAF* B-raf and v-raf murine sarcoma viral oncogene homologue B1, *NA* not available

## Biomarkers for PD-1/PD-L1 blockade therapies

**Fig. 2:**
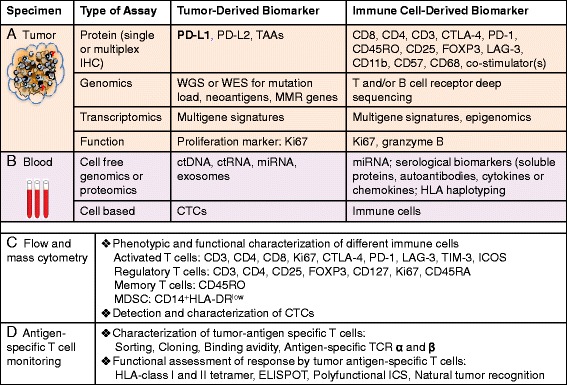
Immune monitoring strategies for patients receiving checkpoint blockage therapy. Technologies that are currently used to assess the potential immune biomarkers. **a** Tumor and immune cells in tumor specimens could be evaluated by immunohistochemical stain (IHC) or immunofluorescence assays, molecular or genetic profiling analysis, and cellular functional assays. The tumor microenvironment can be dissected histopathologically to characterize spatial relationships between tumor and immune infiltrates. Transcriptional profiling assays can evaluate changes in gene expression in both the tumor cells and lymphocytes. Deep sequencing techniques enable quantification of changes in individual T/B cell clonotypes. **b** Peripheral blood provides a minimally invasive way to allow serial monitoring of dynamic changes of immune biomarkers during cancer immunotherapy. The analysis of changes in cell counts with therapy, changes in cytokine levels, circulating tumor cells, tumor-derived nucleotides, and immune cells. **c** Flow cytometric analysis of TILs anPBMCs for quantitating the effect of therapy on immune subsets such as activated CD8 + PD1+ T cells, CD4 + FOXP3 + CD25^hi^ Tregs, or myeloid-derived suppressor cells. Using polychromatic flow cytometry, multiple surface and intracellular markers can be detected, allowing in-depth characterization of T cell phenotype and activation state. **d** Multifunctional flow T cell assay, MHC tetramer staining and ELISPOT can be used to analyze the presence and function of tumor-specific T cell subpopulations. *Abbreviations*: *PD-L1* programed death-1, *IHC* immunohistochemistry, *ELISPOT* enzyme-linked immunospot assay, *CTCs* circulating tumor cells, *WES* whole exome sequencing, *NGS* next-generation sequencing, *TIM-3* T cell immunoglobulin domain and mucin domain, *LAG* lymphocyte-activation gene, *ICOS* inducible costimulators, *MDSC* myeloid-derived suppressor cells, *HLA* human leukocyte antigen

Translational biomarker research is critical for the future of drug development for clinical immune checkpoint blockade therapy. Only one PD-L1 IHC has been approved as a companion diagnostic by the US FDA for patients with advanced NSCLC for pembrolizumab [68]. The challenges in developing immuno-oncology biomarkers for clinical use have been recognized by regulatory agencies and professional societies such as the Society for Immunotherapy of Cancer (SITC) [69]. Over the past several years, the SITC Immune Biomarkers Task force has provided the roadmap and regular updates on immune biomarker development [70–72]. Immunological changes in peripheral blood and tumor could potentially reflect tumor response in patients and serve as immune biomarkers. The immune monitoring assays had been developed to evaluate these potential biomarkers before and after immune checkpoint blockade therapies and investigate their correlation with clinical outcome (Fig. 2). These biomarkers could be classified into two groups: tumor-derived immune biomarkers and immune cell-derived biomarkers as discussed in the following sections. Table 3 summarizes currently available quantitative biomarker assays for immune checkpoint inhibitors.

**Table 3:** Currently available translational biomarker assays for immune checkpoint inhibitors

Biospecimen	Method	Tissue/cell types	Pros	Cons	References and recommended reading
FFPE	IHC	Tumor cells or tumor infiltrating immune cells	Direct detection; accurately pinpoint cancer cells; highly sensitive; simplicity; low cost	Requirement of trained pathologists; inconsistency for criteria used to score tumors such as PD-L1-positive or negative	Herbst R (2014) [145]; Loughlin PA (2007) [164]
	Multicolor IHC	Tumor cells or tumor infiltrating immune cells	Broad dynamic range; capability for multiplexing using different fluorescence channels; >10 protein targets are identified in the same sample; amenability for co-localization studies	Absence of rigorous quantitative tests; limitation in some biomarker-driven clinical trials; user must select combinations of dyes	Carvajal-Hausdorf DE (2014) [165]
	T cell receptor deep sequencing	TILs	T cell count information; T cell clonality in tumor	Heterogeneous expression of TIL	Robbins HS (2013) [100]
	Whole exome sequencing (WES)	Tumor cells	Characterization of tumor mutation load including nucleotide substitutions; structural rearrangements and copy number alterations; identification of the neoantigens and neoepitopes; affordable cost	Require high-performance deep sequencing, computational bioinformatics support; The pipelines are still at early developmental phase	Snyder A (2014) [104]; Rizvi NA (2015) [105]; Bouffet E (2016) [107]; Chen K-H (2016) [166] Hugo W (2016) [106]
Blood	ELISPOT assays (IFNγ and granzyme B)	T cells in PBMCs	Detection of tumor antigen-specific CD4^+^ and CD8^+^ T cell response with good assay sensitivity; Relatively well validated assay	A poor correlation with clinically relevant immune responses	Shafer-Weaver K (2006) [167]; Janetzki S (2008) [95] Malyguine A (2012) [94]; Janetzki S (2015) [93];
	Flow cytometry (tetramer, polyfunctional analysis)	T cells in PBMCs	Assessment of tumor antigen-specific CD4^+^ and CD8^+^ T cells response; measure multiple functions; detection of neoantigen-specific CD8 + PD-1+ T cells; minimally invasive	Merely in lab research, not as routine clinical monitoring yet	Yuan J (2008) [84]; Attic S (2011) [85]; McNeil LK (2013) [86]; Barrera L (2015) [168]; Gros A (2016) [118]
	Flow cytometry phenotype staining	Whole blood immune phenotype	Analyses of the frequency and proliferation of different subsets of immune cells; routine operation	Dedicated resource and staff to perform the analyses	Streitz M (2013) [169]; van Dongen JJ (2012) [170]
	RNA-Seq (NGS)	T cells in PBMCs	Identification of genetic variants; a broader dynamic range; detection of more differentially expressed genes; fast and high efficiency	More expensive than microarray; more complex for analysis; bulk signature, not single cell signals; need more validation	Zhao S (2010) [171]
	qPCR assay	T cells in PBMCs	High specificity; able to detect the reactivity of low-frequency T cells in the peripheral blood of metastatic cancer patients	Bulk signature, not single cell signals; need more validation	Kammula US (2008) [172]
	Flow cytometry	CTCs	Qualitative analysis at the single cell level in a relatively short period of time; decrease the amount of blood needed; provide valuable information regarding the frequency, phenotype and/or the functionality of T cells	Expensive; need more validation	Zaritskaya L (2010) [83]
	Cell sieve microfiltration assay and QUASR technique	CTCs	PD-L1 levels from CTCs or CAMLs serves as a surrogate for PD-L1 expression in tumor; as a marker for immunotherapy response	Limited in lab research; need more validation	Steven HL (2015) [173]; Adams DL (2014) [174]

*Abbreviations*: *FFPE* formalin-fixed paraffin-embedded, *PD-L1* program death-1, *TIL* tumor-infiltrating lymphocytes, *IHC* immunohistochemistry, *CAMLs* cancer-associated macrophage-like cells, *ELISPOT* enzyme-linked immunospot assay, *CTL* cytotoxic T-lymphocytes, *CTCs* circulating tumor cells, *PBMC* peripheral blood mononuclear cell, *WES* whole exome sequencing, *NGS* next-generation sequencing

### Current immune monitoring assays

#### IHC staining

PD-L1 is the best studied immuno-oncology biomarker to date. Currently, PD-L1 IHC using 22C3 antibody is the only FDA-approved companion diagnostic for selecting NSCLC patients for pembrolizumab [68]. Table 4 summarizes the IHC assays used for PD-L1 expression using baseline archival tumor specimens in these clinical studies [73]. There are many variables in these IHC assays. First, the time between sample collection and treatment with a PD-1 or PD-L1 inhibitor is not controlled. Second, tumor cells, immune cells, as well as stroma cells can express PD-L1 with considerably heterogeneity within the tumor microenvironment. Third, PD-L1 expression is induced by IFNγ during disease progression and treatment. Fourth, different PD-L1 antibodies were used for different immune checkpoint blockade clinical studies. Currently, there is no validated antibody for IHC staining for this class of immune checkpoint inhibitors—each sponsor uses different antibodies. Antibodies staining tumor cells or stroma cells may have different PD-L1 epitopes (Table 4). An additional challenge is that none of the commercial PD-L1 IHC test has been validated in terms of clinical outcomes. Head-to-head comparisons among three different commercially available PD-L1 assays (Dako 22C3, Dako 28-8, and Ventana SP263 as the reference) using 500 formalin-fixed, paraffin-embedded archival samples of NSCLC tissue obtained from commercial sources found 91 to 95 % correlation, which is comparable to typical within assay agreement for IHC across the dynamic range at multiple cutoffs [74]. A recent meta-analysis demonstrates that PD-L1 expression is significantly associated with clinical response to anti-PD-1/PD-L1 antibodies in patients with malignant melanoma (MM) or non-squamous NSCLC. However, a proportion of PD-L1-negative patients also benefits from anti-PD-1 therapy in MM and squamous-NSCLC. Thus, expression of PD-L1 in tumor tissues cannot be used as a predictive biomarker of eligibility for treatment with anti-PD-1/PD-L1 antibodies. It may represent a correlation marker for non-squamous NSCLC and melanoma [75].

**Table 4:** Immunohistochemistry assays for PD-L1 expression

Drug	Antibody (marker)	Rx line	Tumor type	Targeted cells	Tumor specimen	Cutoff point (%)	ORR % in IHC+ cases (95 % CI)	ORR % in IHC− cases (95 % CI)	Predictive role	*P* value	References
Nivolumab	28-8 rabbit (Dako)	1 L	Melanoma	TCs	Archival FFPE or new biopsy	5	58 (46,69)	41 (35,48)	Yes	0.001	Larkin J (2015) [130]
Nivolumab + ipilimumab							72 (60,82)	55 (48,62)	Yes	0.001	
Nivolumab		≥2 L				5	44 (30,58)	20 (11,32)	Yes	NA	Weber JS (2015) [128]
Nivolumab + ipilimumab		1 L				5	58 (37,78)	55 (42,69)	No	>0.05	Postow MA (2015) [4]
Nivolumab		1 L				5	53 (41,64)	33 (25,42)	No	>0.05	Robert C (2015) [129]
		≥2 L	NSCLC	TCs	Archival FFPE or new biopsy	1	31 (23,40)	9 (5,16)	Yes	0.002	Paz-Ares L (2015) [156]; Updated in Borghaei H (2015) [126]
						5	36 (26,46)	10 (6,17)	Yes	0.002	
						10	37 (27,48)	11 (6,17)	Yes	0.002	
		≥2 L			Archival FFPE	1	17 (9,29)	17 (8,29)	No	0.9364	Brahmer JR (2015) [125]
						5	21 (10,37)	15 (8,25)	No	0.2908	
						10	19 (8,36)	16 (9,26)	No	0.6411	
		≥2 L			Archival FFPE	1	20 (5,35)	13 (2,28)	No	>0.05	Rizvi NA (2015) [131]
						5	24 (5,43)	14 (3,25)	No		
		1 L			Archival FFPE	5	31 (NA)	10 (NA)	No	>0.05	Gettinger SN (2015) [157]
Nivolumab + ipilimumab		1 L			Archival FFPE	5	19 (NA)	14 (NA)	No	>0.05	Antonia SJ (2014) [158]
Nivolumab	5H1 and anti-PD-1 monoclonal M3	≥2 L			Archival FFPE	5	39 (34,44)	6 (1,12)	Yes	0.025	Taube JM (2014) [113]
Pembrolizumab	22C3 mouse (Dako)	≥1 L	NSCLC	TCs and ICs	New biopsy	50	45 (33,57)	17 (10,25)	Yes	0.001	Garon EB (2015) [5]
		1 L			New biopsy	50	47 (23,72)	19 (8,38)	Yes	NA	Rival NA (2015) [159]
		Any			Archival FFPE	1	25 (NA)	13 (NA)	Yes	NA	Garon EB (2014) [160]
		≥1 L			Archival FFPE	50	30 (23,39)	9.8 (NA)	Yes	NA	Herbst RS (2015) [140]
							29 (22,37)	10.7 (NA)			
		1 L			Archival FFPE	50	16 (NA)	10 (NA)	Yes	NA	Garon EB (2014) [161]
Atezolizumab (MPDL3280A)	SP142 rabbit (Roche Ventana)	≥2 L	NSCLC	TCs and ICs	Archival FFPE and new biopsy	50	45 (23,68)	14 (6,25)	Yes	NA	Leora H (2015) [162]
		≥2 L	NSCLC			1+	31 (25,37)	20 (14,26)	Yes	0.015	Herbst RS (2014) [145]
			Solid Tumor			1+	29 (27,31)	13 (10,16)	Yes	0.007	
		≥2 L	NSCLC			2+	18 (NA)	8 (NA)	Yes	NA	Spira AI (2015) [143]
Durvalumab (MEDI-4736)	SP263 rabbit (Roche Ventana)	≥2 L	NSCLC	TCs	Archival FFPE and new biopsy	25	39 (NA)	5 (NA)	Yes	NA	Segal NH (2014) [163]
							33 (13,59)	30 (16,47)	No for combo	NA	Antonia SJ (2016) [155];
							22 (3, 60)	29 (8, 58)	No for monotherapy	NA	

*NA* not available

The presence of tumor-infiltrating lymphocytes (TILs) at tumor microenvironment has been associated with an improved clinical outcome [76–78], regardless of cancer treatment. TIL counts are primarily measured by IHC [79]. Recent development of multicolored immunohistochemistry staining resulted in a multiplex of up to seven fluorescent dyes effectively interrogated in microscopes equipped with a multispectral camera [80]. The multispectral fluorescent IHC with a panel including CD3, CD8, FoxP3, CD163, and PD-L1 was used to quantify the density of TIL subpopulation in advanced melanoma patients.

#### Flow cytometry staining

Flow cytometry traditionally uses fluorochrome-labeled antibodies to identify cellular protein expression of the target cells. Flow cytometric analysis of peripheral blood mononuclear cells (PBMCs) and TILs can quantitate the effect of therapy on low-frequency immune subsets such as Treg, MDSC, and activated CD8^+^ T cells [81]. The biggest advantage of flow cytometry is the combination of multiparameter measurements in a high-throughput manner. With recent advances in laser and fluorochrome technology, commercially available systems allow for detection of up to 18 colors at analysis rates of more than 20,000 events per second. This tremendous power allows for the analysis of multiple characteristics of rare cells such as tumor antigen-specific T-lymphocytes, including their phenotype and functional activity [82]. Flow cytometric assays are currently widely used for the enumeration and phenotypic characterization of lymphocytes, measuring cytokines, secreted molecules, intracellular signaling, function, and cell proliferation [83]. Using polychromatic flow cytometry, multiple surface and intracellular markers can be detected, allowing in-depth characterization of T cell phenotype and activation state [84]. In addition to flow cytometry, antigen-loaded soluble major histocompatibility complex (MHC) tetramer stains can be used to analyze tumor-specific T cell subpopulations [82]. This tetramer/pentamer staining has been used for phenotypic and quantitative analysis of antigen-specific CD8 T cells [85, 86]. The human leukocyte antigen (HLA) system is a gene complex encoding the MHC proteins in humans. HLA-peptide complexes binding to T cell receptors could further define the specificity for epitopes and alleles [87]. Recently, mass cytometry or CyTOF which replace the fluorescent labels with heavy metal ions is under investigation for its application in immune biomarker research [88].

#### ELISA and protein microarray

Biomarkers in the blood have been pursued because specimens are easy to collect and assays are available for serial monitoring and quantitative measurements [89]. Enzyme-linked immunoabsorbent assay (ELISA) is widely used to assess the production of soluble mediators such as cytokines, chemokines, and tumor antigen-specific antibodies in peripheral blood before and after treatment. Novel protein microarray technologies such as ProtoArray® are available to analyze the serological response of up to 9000 proteins simultaneously [90–92].

#### ELISPOT

Enzyme-linked immunospot (ELISPOT) assays are robust and standardized tests that quantify both the frequency and function of T or B cells at the single cell level. Both PBMCs and isolated CD4^+^ and CD8^+^ T cell subsets could be stimulated by antigens and cultured in a 96-well plate with a nitrocellulose membrane coated with antibodies for cytokines of interest. These assays, such as the IFNγ ELISPOT, have gained increasing popularity for monitoring clinical trials and in basic research [93–95]. Results from various clinical trials, including peptide and whole tumor cell vaccination and cytokine treatment, showed the suitability of the IFNγ ELISPOT assay for monitoring T cell response [96]. ELISPOT assays revealed that 75 % of melanoma patients vaccinated with antigenic peptide demonstrated specific immune responses [97, 98]. Besides IFNγ, other cytokines such as IL-2, IL-5, IL-10, IL-17, granzyme B, TNF, and granulocyte-macrophage colony-stimulating factor (GM-CSF) are commonly measured by ELISPOT assays. It has been expanded to more than 2~3 parameters with the introduction of fluorescent dyes [99].

#### T cell or B cell receptor deep sequencing

To develop a robust, quantitative method with high reproducibility, Robins et al*.* [100] uses a digital droplet PCR technique call a Quan*TIL*fy assay. High-throughput quantitative sequencing of the rearranged TCR β genes uses the ImmunoSeq assay. The TCR loci undergo somatic gene rearrangements in the 52 variable (V), diversity (D), and 13 joining (J) regions of the β chain and variable (V) and joining (J) regions of α chain. There are 52 Vβ segments and 13 Jβ segments that can rearrange. With the right TaqMan probes-DNA from T cells can be easily identified with reference to total DNA, as was shown when purified human T cells were obtained from a healthy donor and then diluted and mixed with human lung fibroblasts at different ratios. This technique can also identify clonal expansion (the mark of the adaptive immune response). TCR deep sequencing has been used to show the correlation between TCR repertoire and clinical response to cancer immunotherapy in patients with melanoma or prostate cancer treated with ipilimumab [101]. Creation of a responder library for TCR repertoire will greatly advance the field, especially for genetically modified TCR cell transfer therapy.

#### WES for mutation load

The cancers that respond best to PD-1 inhibitors, such as advanced melanoma and NSCLC, are the tumor types that are genetically very complex with a high nonsynchronous mutation load. This discovery led to an interest in neoantigens (i.e., tumor-specific mutant antigens (TSMAs)) that are immunogenic [102]. The advances in both affordable next-generation sequencing (NGS) technology and bioinformatics make it feasible to assess the full mutation load in individual tumors and to identify neoantigens by comparing WGS of tumor and normal tissues [103]. Computation prediction algorithms and the tandem mini-gene library allow for the evaluation of the immunogenicity of both CD4^+^ and CD8^+^ T cell neoepitopes. Mutation load and neoepitopes have been explored for their correlation with clinical outcomes in cancer patients treated with the PD-1/PD-L1 immune checkpoint blockade [104–107]. However, whole exome sequencing (WES) currently is not used in routine clinical practice. The role of tumor load assessed in targeted exome sequencing (TES) assays in predicting response to immune checkpoint inhibitors remains to be determined.

#### Genetic susceptibility

Genetic factors are known to modulate the immune response to a therapeutic protein product. In particular, some HLA haplotypes may predispose patients to developing either exceptional or undesirable antibody responses to immune checkpoint blockage therapies [108]. If both are appropriate and feasible, HLA mapping studies may help define a subset of patients who are at increased risk. Moreover, genetic polymorphisms in cytokine genes may upregulate or downregulate immune responses [109], which is why the FDA recommends that researchers evaluate the genetic factors that may modulate the immune response to a therapeutic protein. For example, the subset of patients who generate neutralizing antibodies to IFNβ products are more likely to possess distinct HLA haplotypes [108]. Thus, knowledge of the heightened susceptibility of patients with such HLA haplotypes may guide us to prevent such responses or pursuit other treatment options (http://www.fda.gov/ucm/groups/fdagov-public/@fdagov-drugs-gen/documents/document/ucm338856.pdf).

### Tumor-derived immune biomarkers

#### PD-L1 expression

Upregulation of PD-L1 expression level has been reported in many different cancer types (e.g., melanoma (40–100 %), NSCLC (35–95 %), and multiple myeloma (93 %)). High levels of PD-L1 expression have been linked to poor clinical outcomes [42, 110, 111]. Until now, anti-PD-L1 IHC on tumor specimens is the most commonly used biomarker for selecting patients who are likely to respond to treatments [89]. Constitutively overexpressed PD-L1 in melanoma is associated with poor clinical outcome [112]. However, the PD-L1 overexpression in the context of CD8-positive cells is associated with a better prognosis [113]. Although PD-L1 IHC using the 22C3 antibody has been approved by the US FDA as the only predictive companion diagnostic for selecting NSCLC patients for pembrolizumab, tumor responses have been seen in low PD-L1-expressing tumors. Conflicting results have been reported with other PD-L1 antibodies and drugs. Table 4 summarizes the reported data of PD-L1 IHC in current clinical trials.

Several tumor characteristics, such as a high mutation load, smoking-related tumors, and tumors with mismatch repair genes are associated with improved objective tumor response rate (ORR), durable clinical benefit (DCB), and PFS to immune checkpoint blockade antibodies. Notably, whole exome sequencing has revealed that a nonsynonymous mutation load of >200 per tumor correlates with clinical responses to PD-1 mAb in NSCLC, melanoma, and colorectal cancer with microsatellite instability (MSI; i.e., mutations in DNA mismatch repair genes) [114–116]. Rizvi and his colleagues showed that mutation load correlates with improved ORR (63 versus 0 %), DCB (73 versus 13 %), and PFS (14.5 versus 3.7 months) [105]. Moreover, the efficacy was associated with a molecular smoking signature. The ORR was 56 and 17 % in transversion-high tumors and transversion-low tumors, respectively; the rate of DCB was 77 versus 22 %, respectively; and the PFS was also significantly longer in transversion-high tumors compared to transversion-low tumors. Efficacy was also connected with higher neoantigen burden, but additional genetic features are likely to play a role in determining the tumor response [116, 117]. Neoantigen-specific CD8+ T cell responses paralleled tumor regression in one responder, implying that anti-PD-1 therapy enhanced neoantigen-specific T cell reactivity [118].

### Immune cell-derived biomarkers

TIL cells have been shown to express significantly higher levels of PD-1 than T cells in normal tissue [119]. The tumor microenvironment may secrete pro-inflammatory cytokines to upregulate the expression of PD-1 on TIL cells to ensure that they can respond to the high levels of PD-L1 expressed on the tumor [120]. Preexisting CD8^+^ T cells distinctly located at the invasive tumor margin are associated with expression of the PD-1/PD-L1 immune inhibitory axis [121]. The proliferation of intratumoral CD8^+^ T cells directly correlated with radiographic reduction in tumor size in metastatic melanoma patients treated with pembrolizumab. Pretreatment samples obtained from responding patients showed higher numbers of CD8^+^ T cells, PD-1, and PD-L1-expressing cells at the invasive tumor margin and inside tumors, with a more clonal TCR repertoire. Using multivariate analysis, researchers established a predictive model based on CD8 expression at the invasive margin and validated the model in an independent cohort of 15 patients. Their findings indicate that tumor regression after PD-1 blockade requires preexisting CD8^+^ T cells that are negatively regulated by PD-1/PD-L1-mediated adaptive immune resistance. In addition, the tumor microenvironment can be dissected histopathologically in order to characterize spatial relationships between tumor and immune infiltrate, and transcriptional profiling assays can evaluate changes in gene expression in both the tumor and in lymphocytes [122].

Given that TIL in the tumor microenvironment affects the prognosis of the tumor, a new TNM Immune system (TNM-I) system has been proposed and is currently being validated by an international consortium [123]. Early studies suggest that activated T cells are not pharmacodynamic markers of nivolumab treatment [124]. It remains to be determined whether nivolumab treatment increases CD8 effector memory cells in cancer patients. With technical advances, detection of CD8-positive, PD-1-positive T cells in the peripheral blood of melanoma patients receiving PD-1 inhibitors is feasible. Furthermore, the tumor-antigen specificities and TCR repertoires of the circulating and tumor-infiltrating CD8^+^PD-1^+^ cells appeared similar [118].

## Conclusions

The era of cancer immunotherapy has arrived. This major breakthrough in cancer treatment holds great promise for increasing cure rates for patients with various cancer types in multiple disease settings. Checkpoint blockade antibodies targeting CTLA-4, PD-1, and PD-L1 axis have demonstrated impressive and durable disease control and promising responses in patients with multiple tumor types. Despite these advances, only a subset of patients benefits from immune checkpoint blockade therapies, with some patients experiencing mechanism-based irAEs. Translational biomarker research is one way to overcome these limitations of therapy. Biomarkers play a critical role in understanding potential mechanisms of action in patients, identifying patients who are likely to respond to the growing list of cancer therapeutics and avoiding the irAEs. Several *tumor-derived biomarkers* have been reported from recent correlative studies including PD-L1 expression on tumor cells, high tumor mutational load, and neoantigens. In addition, several *immune cell-derived biomarkers* showed better correlation with the clinical outcomes. The presence of TILs in tumor microenvironments, increased PD-L1 expression on immune cells, and the ratio of effector CD8^+^ T cells to FoxP3^+^ regulatory T cells in tumors are some examples of markers for clinical outcomes. Future studies are warranted to harmonize companion diagnostics (such as PD-L1 IHC) for accurate clinical assessment and application of the class of PD-1/PD-L1 inhibitors. It is of importance to further evaluate mutation load as a potential biomarker and develop effective tumor antigen-specific T cell assays to differentiate immunogenic neoepitopes from putative ones. The advances in translational immune biomarker research are essential for personalized cancer immunotherapy as either monotherapy or combinational therapy.

## Abbreviations

AKT, protein kinase B; APCs, antigen-presenting cells; BRAF, B-raf and v-raf murine sarcoma viral oncogene homologue B1; BTLA, B- and T-lymphocyte attenuator; CAMLs, cancer-associated macrophage-like cells; CEACAM, carcinoembryonic antigen-related cell adhesion molecule; CTCs, circulating tumor cells; CTL, cytotoxic T-lymphocytes; CTLA-4, cytotoxic T-lymphocyte-associated protein 4; DC, dendritic cell; DLT, dose-limiting toxicity; EGFR, epidermal growth factor receptor; ELISPOT, enzyme-linked immunospot assay; FFPE, formalin-fixed paraffin-embedded; GITR, glucocorticoid-induced tumor necrosis factor receptor; GM-CSF, granulocyte-macrophage colony-stimulating factor; HLA, human leukocyte antigen; HVEM, herpes virus entry mediator; ICOS, inducible costimulators; IDO, indoleamin 2,3-dioxygenase; IHC, immunohistochemistry; IL-2R, IL-2 receptor; irAEs, immune-related adverse effects; JNK, c-Jun N-terminal kinase; LAG, lymphocyte-activation gene; MDSCs, myeloid-derived suppressor cells; MEK/ERK, mitogen/extracellular signal regulated kinase; MHC, major histocompatibility complex; NGS, next-generation sequencing; NFκB, nuclear factor kappa-light-chain-enhancer of activated B cells; NSCLC, non-small-cell lung cancer; PBMCs, peripheral blood mononuclear cells; PD-1, programed death-1; PD-L1, programed cell-death ligand 1; PI3K, phosphatidylinositol 3-kinase; q2w, every 2 weeks; q3w, every 3 weeks; q4w, every 4 weeks; QD, once daily; STAT, signal transducer and activator of transcription; Teff, effector T cell; TGF-β, transforming growth factor beta; TIGIT, T cell immunoreceptor with Ig and ITIM domains; TIL, tumor-infiltrating lymphocytes; TIM-3, T cell immunoglobulin domain and mucin domain; TNF, tumor necrosis factors; Treg, regulatory T cells; TSMA, tumor-specific mutant antigen; VEGF, vascular endothelial growth factor; WES, whole exome sequencing
